# Polysaccharides Extraction from *Opuntia milpa alta* and Their Protective Effect on Alcohol-Induced Neuro 2a Cell Damage via Ferroptosis

**DOI:** 10.3390/foods15020249

**Published:** 2026-01-09

**Authors:** Congyue Xu, Lin Chen, Qin Ru, Yuxiang Wu

**Affiliations:** Institute of Intelligent Sport and Proactive Health, Department of Health and Physical Education, Jianghan University, Wuhan 430056, China

**Keywords:** ferroptosispolysaccharides, *Opuntia milpa alta*, Neuro 2a cell, alcohol, ferroptosis

## Abstract

Alcohol enters the brain through the blood–brain barrier and causes neuronal damage in various ways, additionally long-term and heavy drinking also leads to both structural and functional changes in the central nervous system. Currently, there is a lack of specific therapeutic approaches for alcohol-induced nerve injury. *Opuntia milpa alta* polysaccharides (MAPs) have various physiological activities such as antioxidant, anti-inflammatory, and neuroprotective effects, but it is not clear how they protect against alcohol-induced nerve injury. In this study, firstly, we structurally characterized homemade MAPs and analyzed the relevance of MAPs in protecting against alcoholic neuronal cell injury and ferroptosis. The results showed that MAPs consisted of nine different monosaccharides and uronic acids. High performance gel permeation chromatography analysis showed that MAPs were homogeneous heteropolysaccharides with an average molecular weight of 8.79 × 106 Da. Fourier infrared spectroscopy showed that they had sulfated pyranopolysaccharides with uronic acids and both α-glycosidic and β-glycosidic bonds were present. Specific signals of these sugars were observed in 1H and 13C NMR spectra. Favorable thermal stability was manifested up to 256 °C. The MAPs had a three-stranded helical structure and a low overall crystallinity. Iron staining showed that alcohol caused significant brown deposition in cells. MAPs significantly ameliorated alcohol-induced cellular damage, reduced iron deposition, and orchestrated the expression of proteins associated with ferroptosis. These results suggest that MAPs protect against alcohol-induced neurological damage, possibly by impeding the onset of cellular ferroptosis.

## 1. Introduction

According to the World Health Organization’s (WHO) Global Burden of Disease study, more than 5% of the world’s burden of disease and cause of death is attributed to alcohol consumption [1]. As the number of people who drink alcohol, the amount, and frequency of alcohol consumption continue to increase, it not only causes various social problems, but also produces a mental withdrawal response and causes damage to various tissues and organs. The central nervous system, as one of the main target organs of alcohol damage, has a complex mechanism of action, which is related to a variety of cytokines, oxidative stress and other factors such as free radical damage [2]. Ferroptosis has emerged as a recently identified form of regulated cell death over the past several years. It has been shown that alcoholic liver injury [3], atrial fibrillation [4] and anxiety/depression [5,6] due to alcohol consumption are associated with ferroptosis, and thus could be a new strategy for the prevention and treatment of alcoholic nerve cell injury.

Natural polysaccharides, a class of biocompatible and biodegradable polymers, are utilized across the food, cosmetic, and pharmaceutical industries because of their therapeutic benefits and favorable safety profile [7]. The specific structural attributes, particularly the constituent monosaccharides and the molecular weight profile, critically govern these biological functions. For example, it has been reported that depolymerization enhances the antioxidant activity of *Opuntia ficus indica* polysaccharides [8]; Zhang and colleagues [9] demonstrated that elevated molecular mass coupled with abundant glycosyl residues in polysaccharides correlates with superior suppression of α-amylase activity.

*Opuntia milpa alta*, a cactus species indigenous to Mexico’s Milpa region, produces edible stems and fruits. This drought-tolerant plant has been introduced globally, with established cultivation across Asia, Europe, and Africa. The genus Cactus has a long history of edible and medicinal uses, and Extracts derived from it have been demonstrated to possess a range of biological activities, including antioxidant [10], antitumor properties [11] and antidiabetic [12]. Among them, the neuroprotective effects of *Opuntia milpa alta* polysaccharides (MAPs) have attracted our attention, as they protect against focal cerebral ischemia, chronic cerebral hypoperfusion, and cerebral oxidative stress injury [13,14,15]. However, the protective effects of MAPs against alcohol-induced neuronal cell injury have not been reported.

This study therefore pursued dual objectives: to characterize the structural properties of *Opuntia milpa alta* polysaccharides (MAPs), to evaluate their neuroprotective efficacy against ethanol-induced neuronal damage, and to propose ferroptosis inhibition as the potential mechanistic basis for MAP-mediated cytoprotection.

## 2. Materials and Methods

### 2.1. Reagents and Materials

Plant specimens of *Opuntia milpa alta* were procured from Fanxing Horticultural Center (Suqian, Jiangsu Province, China) and subsequently authenticated by co-author Prof. Lin Chen; Cellulase (50 U/mg) and pectinase (30 U/mg) are both food grade and purchased from Beijing Solarbio Science & Technology Co., Ltd. (Beijing, China); ethanol, trifluoroacetic acid, methanol, 3-Methyl-1-phenyl-2-pyrazolin-5-one (PMP), hydrochloric acid, chloroform, Dimethyl sulfoxide (DMSO) were procured from Sinopharm chemical reagent Co., Ltd. (Shanghai, China); Monosaccharide standards (D-Glucuronic acid, D-Galacturonic acid monohydrate, L-Rhamnose, L-Arabinose, D-Mannose, D-Galactose, D-Ribose, D-Xylose and D-Glucose)were used Shanghai yuanye Bio-Technology Co., Ltd. (Shanghai, China); Anti-ferroportin-1 (FPN-1), Anti-glutathione peroxidase 4 (GPX4), and Anti-transferrin receptor (TFRC), anti-acyl-CoA synthetase family member 4 (ACSL4), anti-4-hydroxynonedal (4-HNE) were from Abcam (Cambridge, UK); Anti-solute carrier family 7 member 11 (SLC7A11), anti-β-actin, goat anti-rabbit-HRP second antibody, The cell viability assay reagent MTT (3-(4,5-dimethylthiazol-2-yl)-2,5-diphenyltetrazolium bromide) were commercially acquired from Beyotime Biotechnology (Haimen, China); Anti-ferritin heavy chain (FTH1) was from Absin Technology (Manchester, UK); Malondialdehyde (MDA) Assay Kits, Superoxide Dismutase (SOD) Assay Kits and L-glutathione (GSH) Assay Kits were obtained from Nanjing Jiancheng Bioengineering Institute (Nanjing, China); Goat anti-mouse IgG was from Wuhan Servicebio Technology Co., Ltd. (Wuhan, China); Penicillin, fetal bovine serum (FBS), streptomycin, Dulbecco’s Modified Easle’s medium /F12 (DMEM/F12), and trypsin were purchased from Thermo Scientific (Waltham, MA, USA); Ferrostatin-1 (Fer-1) was purchased from Med Chem Express Ltd. (Monmouth Junction, NJ, USA); Total Iron and Ferrous Iron Assays were quantified using specific colorimetric assays (Biomedical Technology Co., Ltd., London, UK).

### 2.2. MAPs Extraction

Based on the literature but modified appropriately [13], the polysaccharides extracted by enzymatic extraction. Fresh stems of *Opuntia milpa alta* underwent sequential processing: thorough washing, spine removal, surface peeling, and mechanical fragmentation into small segments prior to extraction. After degreasing and depigmentation, it was dried and sealed for preservation. The reaction system for enzymatic extraction was composed of 50 g *Opuntia milpa alta* powder suspended in 1.5 L of citrate-phosphate buffer (0.130 M, pH 3.5), corresponding to a solid-to-liquid ratio of 1:30 (g/mL). Enzymes were then added to achieve final concentrations of 0.3% (*w*/*w*) cellulase and 0.7% (*w*/*w*) pectinase relative to the plant material. The enzymatic digestion proceeded under constant stirring at 40 °C for 90 min. Following reaction termination via 10 min boiling water bath treatment, centrifugation (4000× *g*, 15 min) yielded the supernatant. This aqueous phase underwent concentration and deproteinization prior to polysaccharide precipitation with 95% (*v*/*v*) ethanol at 4 °C for 12 h. Final purification was achieved by DE-52 cellulose column chromatography. The polysaccharides prepared by enzymatic extraction had 10.14% extraction yield.

### 2.3. Chemical Composition and Structural Characterization

#### 2.3.1. Chemical Characterizations

Neutral sugar quantification employed the phenol-sulfuric acid method with L-arabinose as the reference monosaccharide [16]. Uronic acid levels were determined via the carbazole-sulfuric acid assay, utilizing D-galacturonic acid for standard curve construction [17].

#### 2.3.2. Monosaccharide Composition Analysis [18]

After hydrolysis and derivatization of MAPs, the monosaccharide fractions and molar ratios of MAPs were detected by precolumn derivatization high performance liquid chromatography. Chromatographic separation was conducted using an Agilent 1260 Infinity II HPLC system (Santa Clara, CA, USA) equipped with an Ultimate™ XB-C18 column (250 × 4.6 mm, 5 μm). Operational parameters included: 20-μL injection volume, 35 °C column temperature, 1.0 mL·min^−1^ flow rate, and 250 nm detection wavelength. The mobile phase composition was as follows: solvent A was pure acetonitrile, and solvent B consisted of 900 mL of ultrapure water, 100 mL of pure acetonitrile, 0.45 g of potassium dihydrogen phosphate and 0.5 mL of triethylamine (pH 6.5). The time gradient was 0~3~8~12~17.5~25~30~35 min, and the corresponding volume fractions were 4%~4%~7%~8.7%~11.4%~11.4%~50%~4% of solvent A.

#### 2.3.3. Molecular Weight Analysis [19]

The relative molecular weight distribution was assessed via gel permeation chromatography (GPC) using a Shimadzu LC-40D instrument (Kyoto, Japan). An OHpak SB-806M HQ analytical column (Shodex, Tokyo, Japan) maintained at 35 °C was employed for separation, with detection achieved by a RID-20A refractive index detector also held at 35 °C. The mobile phase consisted of an aqueous 0.71% (*w*/*v*) sodium sulfate solution delivered isocratically at 0.6 mL/min. Prior to analysis, the polysaccharide was dissolved in the mobile phase, passed through a 0.22 μm syringe filter, and a 10 μL aliquot was introduced into the system. For system calibration, a series of dextran standards with certified peak molecular weights (Mp) of 2.7, 5.25, 9.75, 13.05, 36.8, 64.56, and 300.6 kDa were utilized. Each standard was prepared at 2 mg/mL in ultrapure water and analyzed under the same chromatographic conditions to generate a calibration curve. MAPs molecular weight parameters were subsequently derived from this calibration.

#### 2.3.4. FITR Analysis [20]

Fourier-transform infrared (FT-IR) spectral analysis of MAPs was performed on a BRUKER TENSOR 27 spectrometer (Karlsruhe, Germany). Approximately 2 mg of dried polysaccharide was homogenized with 200 mg spectroscopic-grade KBr and pressed into transparent pellets. Sixty-four scans were co-added per spectrum under nitrogen purge to minimize atmospheric interference. Spectra were acquired across the 4000–400 cm^−1^ range at 4 cm^−1^ resolution using KBr pellet methodology.

#### 2.3.5. NMR Spectroscopy Analysis [21]

For nuclear magnetic resonance (NMR) characterization, approximately 30 mg of the polysaccharide sample was dissolved in 0.55 mL of deuterium oxide (D_2_O, 99.9% atom D). ^1^H and ^13^C NMR spectra were recorded on a Bruker AVANCE III HD superconducting NMR spectrometer operating at a proton frequency of 400 MHz. All measurements were performed at a probe temperature of 298 K.

#### 2.3.6. XRD Analysis [22]

MAPs were dried and ground into fine powder. Appropriate amount of the sample powder was taken and the sample powder was spread evenly on a glass slide and left to dry naturally. The prepared samples were placed in an X-ray diffractometer (X’pert powder, Panalytical, Almelo, The Netherlands) and the diffraction angle 2θ ranged from 5° to 55° was measured.

#### 2.3.7. Helical Structure Analysis [23]

The Congo red method was used to analyze whether MAPs have a three-stranded helical structure. To 1.0 mL of polysaccharide solution (1 mg/mL), 1.0 mL of Congo red solution (0.1 mg/mL) was added and mixed thoroughly. A certain amount of NaOH solution (2.0 mol/L) as well as the corresponding volume of distilled water were added so that the final concentration of NaOH in the solution was 0, 0.1, 0.2, 0.3, 0.4, and 0.5 mol/L, respectively, and the final total volume of the solution was 500 μL, and the solution was allowed to stand for 20 min at room temperature. Absorption spectra from 300 to 700 nm were recorded on a Shimadzu UV-2600i instrument (Tokyo, Japan) at different NaOH molarities. All measurements were referenced against a distilled water blank. The relationship between NaOH concentration and the resultant maximum absorption wavelength was then graphically represented, with molarity as the abscissa and wavelength as the ordinate.

#### 2.3.8. The Thermogravimetric Analysis [24]

The thermal stability of the cactus fruit polysaccharide was evaluated by thermogravimetric analysis (TGA). Approximately 3 mg MAPs was weighed and sealed in an aluminum crucible, with an empty crucible serving as a reference. The analysis was performed under a nitrogen atmosphere with a purge flow of 40 mL/min. The temperature was increased from 40 to 700 °C at a constant heating rate of 10 °C/min.

### 2.4. Cultures of Neuro 2a (N2A) Cells

At 37 °C in a 5% CO_2_ incubator, N2A cells—a mouse neuroblastoma cell line sourced from the Kunming Institute of Cell Library—were cultivated in DMEM/F-12 medium containing 10% FBS and 1% penicillin-streptomycin. Subsequently, the cells were harvested and plated into 24-well plates at 3 × 10^5^ cells per well.

### 2.5. Determination of Cell Viability and Biochemical Indicators: SOD Activity, MDA, GSH, Total Iron and Ferrous Iron Levels

Cell viability was assessed via the MTT assay. In brief, 96-well plates were seeded with cells at 5 × 10^3^ cells/well, and the cells were allowed to attach overnight. Subsequently, they were exposed to varying ethanol concentrations (200–1000 mmol/L) for 24 h. Thereafter, each well received twenty microliters (20 μL) of MTT solution at a concentration of 0.5 mg/mL, with subsequent incubation for 4 h period. After aspirating the supernatant, 150 μL dimethyl sulfoxide (DMSO) was introduced to solubilize the formazan crystals that had precipitated. A microplate reader was used to detect the absorbance at 570 nm. Three biological replicates were performed for the MTT assay, and six technical replicate wells were set for each treatment group in each independent biological replicate. Meanwhile, in cell lysates, commercially available kits were used to determine the concentrations of biochemical indicators following the manufacturers’ recommended instructions [25].

### 2.6. Iron Staining

Perl’s Prussian blue staining was implemented to evaluate iron content, following the method described elsewhere [26]. For this purpose, cell slides were incubated with a freshly mixed solution of 4% HCl and 4% potassium ferrocyanide (1:1, *v*/*v*) for 30 min at 25 °C to form Prussian blue complexes. Following three rounds of washing with PBS, the slides were submerged in methanol supplemented with 1% hydrogen peroxide for a 20 min duration. After undergoing another three PBS washes, the slides were then incubated with 3,3-diaminobenzidine (DAB) until the appearance of brown granules was visualized. The reaction was halted using distilled water, and the processed slides were finally mounted with neutral gum.

### 2.7. Western Blot Analysis

Total protein was isolated from N2a cells by employing RIPA lysis buffer fortified with both protease and phosphatase inhibitors. Protein quantification was performed using a bicinchoninic acid (BCA) kit. Following this, aliquots containing 10 μg of protein from each lysate were subjected to SDS-PAGE separation and subsequent electrophoretic transfer onto nitrocellulose membranes. To reduce nonspecific binding, membranes were first blocked for 1 h using 5% non-fat dried milk at 25 °C. Following this, they were exposed to targeted primary antibodies at 4 °C for 12 h before application of a complementary HRP-conjugated secondary immunoglobulin. The primary antibodies included those against: TFRC, SLC7A11, FTH, FPN, ACSL4, 4-HNE, β-actin (all at 1:1000 dilution), and GPX4 (1:4000). The resulting bands were documented employing the BG-gdsAUTO710MINI detection system (BAYGENE, Beijing, China).

### 2.8. Statistical Analysis

All values are reported as the mean ± standard error of the mean (S.E.M.). Statistical analysis was performed using one-way ANOVA followed by a Tukey post hoc test in GraphPad Prism (version 8.0), with a probability level of *p* < 0.05 considered to indicate statistical significance.

## 3. Results and Discussion

### 3.1. Chemical Composition and Structural Characterization

**Figure 1:**
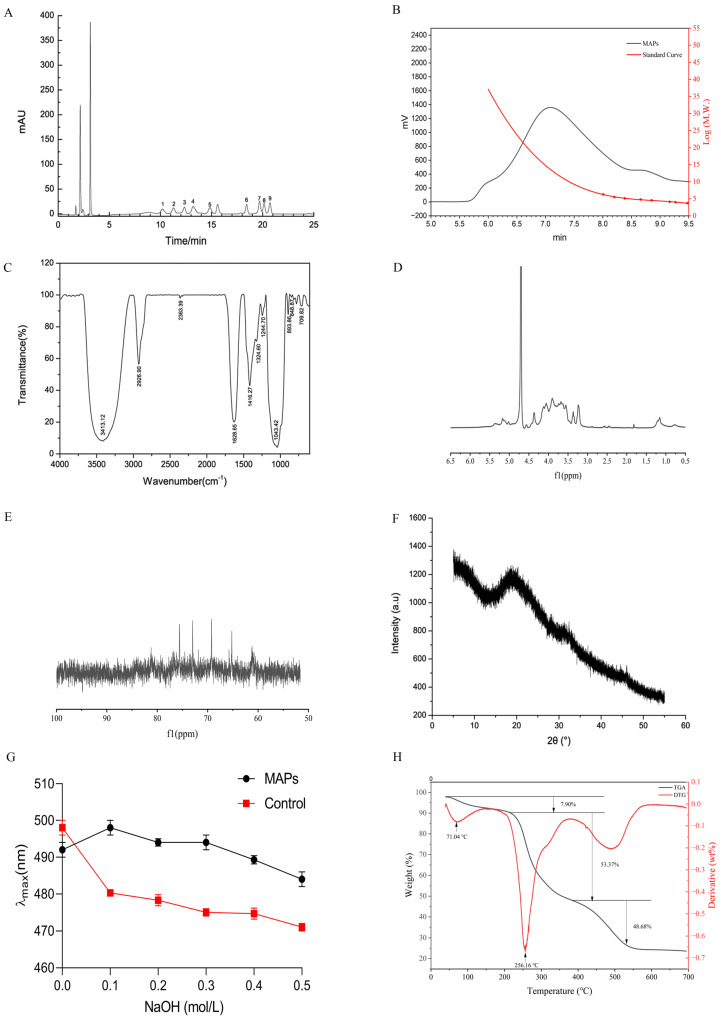
Structural characterization of *Opuntia milpa alta* polyaccharides. (**A**) The monosaccharide composition of MAPs by HPLC. (**B**) The molecular weight of MAPs (**C**) FT-IR spectra of MAPs. (**D**) ^1^H NMR spectrum of MAPs. (**E**) ^13^C NMR spectrum of MAPs. (**F**) The XRD of MAPs. (**G**) Helical structure of MAPs. (**H**) TGA thermograms of MAPs.

Compositional analysis determined that neutral sugars and uronic acids accounted for 74.50% and 11.43% of the polysaccharide solution, respectively. The monosaccharide profile of MAPs, detailed in Table 1 and Figure 1A, was characterized by seven neutral sugars (including mannose, ribose, rhamnose, glucose, xylose, galactose, and arabinose) and two glycuronic acids. The markedly higher levels of arabinose and galactose suggest the potential existence of rhamnogalacturonan-I regions in the polymer structure [8]. The monosaccharide composition of MAPs is affected by cactus species, extraction methods, purification column materials, etc. For example, Chen Y. et al. [13] found that MAPs extracted by water contained the above seven monosaccharides (the largest percentage of which was galactose at 38.25%) by gas chromatography, but two glucuronic acids were not detected. An aqueous extraction protocol was employed by Habibi et al. to obtain water-soluble polysaccharides from the peel of *Opuntia ficus-indica* prickly pear fruits [27], and found that the polysaccharides were mainly composed of galacturonic acid, arabinose and galactose. It indicated that more types of monosaccharides can be obtained by using enzymatic preparations MAPs. The molecular weight of MAPs and dextran standard curve were determined by gel permeation chromatography (GPC) as shown in Figure 1B. The obtained regression equation: y = −1.208517x^3^ + 32.29872x^2^ − 288.7308x + 867.7575, with a high correlation coefficient (R^2^ = 0.9933). Based on this calibration, the Mw of MAPs was calculated to be 8.79 × 10^6^ Da. Based on this composition and the standard residue masses of the constituent monosaccharides, we calculated an average residue molecular weight of approximately 153.6 Da. Consequently, the estimated degree of polymerization (DP) is about 57,200. From this, the approximate number of each monosaccharide residue per average polysaccharide molecule was derived.

By infrared spectroscopy detection, the possible types of a certain spectral band can be analyzed based on the peak area of the absorption peaks, and information such as the type of glycosidic bond, the type of glycan ring, and the substituent characteristics of the polysaccharide samples can be obtained [28]. As shown in Figure 1C, MAPs have characteristic spectral bands for polysaccharides: 3600–3200, 3200–2800, 1400–1200, and 1200–1000 cm^−1^. The FT-IR spectrum displayed characteristic vibrational bands of polysaccharides. A broad peak at 3413.12 cm^−1^ arises from O-H stretching [29], and another distinct peak observed at 2926.90 cm^−1^ indicates C-H stretching vibrations [30]. The strong absorption peak at 1628.65 cm^−1^ was attributed to COOH in GalA, and the presence of COOH was also evidenced by the absorption band at 1416.27 cm^−1^ [31]. These characteristic absorption peaks of saccharides indicated that the MAPs were acidic polysaccharides. The S=O stretching vibration peaks at 1244.70 cm^−1^ for -O-SO3 and C-O-S stretching vibration peaks at 817.17 cm^−1^ indicate that the polysaccharides contain sulfate groups [32], further suggesting that the MAPs are sulfated polysaccharides containing glyoxylates. Absorption between 1200 and 1000 cm^−1^ suggests the presence of a pyranose ring [33], and absorption peaks at 1043.42 cm^−1^ for MAPs are C-O-C asymmetric stretching vibration and C-O stretching vibration indicating that the MAPs are pyranose polysaccharides. The (891 ± 7) cm^−1^ is a β-type isomer caused by the C-H angular vibration, and α-type pyranose is often found in the (844 ± 8) cm^−1^ peak [34], and the absorption peaks at 893.86 cm^−1^ and 848.87 cm^−1^ of the MAPs indicate that both α-glycosidic and β-glycosidic bonds are present.

NMR analysis provided key insights into the structural features of MAPs, including anomeric configuration, glycosidic linkages, and sugar ring conformation [35]. In the ^1^H NMR spectrum (Figure 1D), the anomeric proton signals were observed within the characteristic region of δ 4.1–5.5 ppm. Specific resonances were identified at δ 4.06, 4.13, 4.37, 5.17, and 5.36, with relative integrals as indicated. Based on the chemical shift criterion—where signals above δ 5.0 ppm correspond to α-configurations and those below δ 5.0 ppm to β-configurations [36]—the ratio of α- to β-anomers was calculated to be approximately 1:1.28 from the integrated peak areas. This proportion differed from previous reports using varied extraction methods [37], which reported ratios ranging from 1:1.5 to predominantly β- or α-forms depending on the extraction protocol.

The ^13^C NMR spectrum further confirmed the structural assignments [38], displaying characteristic signals between δ 60–110 ppm. Key resonances were observed at δ 61.24, 65.22, 69.26, 72.97, 75.58, and 103.16 ppm (Figure 1E). Notably, signals in the region of δ 90–110 ppm corresponded to anomeric carbons, while the absence of signals between δ 82–84 ppm indicated the absence of furanose rings, consistent with a pyranose ring conformation for all sugar residues [39]. This observation aligned with the FT-IR analysis. Additionally, signals in the δ 60–80 ppm region were attributed to C2–C6 of the sugar rings, with the resonance at δ 61.24 ppm likely corresponding to the unsubstituted C-6 of mannosyl residues.

X-ray diffraction (XRD) technique is an important analytical method to characterize the structure of macromolecules, which can sensitively detect the amorphousness or crystallinity of samples. In polysaccharides, amorphousness and crystallinity can directly affect a variety of physical properties such as solubility, elasticity, and solubility [40]. Although plant polysaccharides themselves do not have a crystal structure, certain microcrystalline structures can be formed in the process of concentration, freeze-thawing and repeated drying, while the formation of these structures has an important relationship with the primary structure of polysaccharides to a certain extent [41], and therefore the microcrystalline structure of plant polysaccharides can be determined by X diffraction to analyze the monosaccharide composition and the potential pattern of the influence of primary structure differences such as glycosidic bond types on the formation of their microcrystal structures.

The XRD of polysaccharides were investigated as shown in Figure 1F. In the diffraction angle 2θ range from 5° to 55°, the diffraction curves of cactus polysaccharides presented an overall low crystallinity. A diffuse peak appeared around 20°, and no other strong absorption peaks appeared, which is consistent with the results of most plant polysaccharides tested [42]. It suggested that the multimeric homogeneity formed by MAPs is relatively high, which may be related to the formation of a regular high level of structure between the plant polysaccharide molecules due to the presence of cross-links. This is further illustrated by the absence of other diffuse peaks in the plots. Overall, although the MAPs underwent several treatments such as concentration, dialysis and lyophilization during the preparation process, it did not lead to an increase in crystallinity and only a small amount of microcrystalline structure was formed, which ensured its better water re-solubility. We also found that cactus polysaccharides can form complexes with Congo red through Congo red experiments, with a three-stranded helical structure (Figure 1G).

Thermogravimetric (TG) and derivative thermogravimetric (DTG) analyses were performed to evaluate the thermal stability and decomposition pattern of MAPs over a temperature range of 40 to 700 °C (Figure 1H). The weight loss profile revealed three distinct stages of thermal decomposition. The initial stage, occurring up to approximately 180 °C, involved a minor mass loss (7.9%), attributable to the evaporation of free and loosely bound water within the polysaccharide matrix [43]. This observation aligns with findings reported for similar polysaccharide materials [44]. A major decomposition event was observed in the second stage, between 220 and 380 °C, which accounted for a substantial weight loss of 53.37%. This phase corresponds to the primary thermal degradation of the polysaccharide chains [45], consistent with the typical pyrolysis range (210–350 °C) documented for cactus-derived polysaccharides [46]. The maximum rate of this degradation, as indicated by the DTG peak, occurred at 256.16 °C. Subsequent weight loss in the third stage (380–530 °C) is associated with the further breakdown of pyrolysis products, including the fragmentation of oligo- and monosaccharides into volatile compounds such as CO_2_ and H_2_O [47]. Beyond 530 °C, the mass change plateaued, yielding a final residual mass of 23.60% at 700 °C. This residue content indicates a relatively high thermal stability compared to some polysaccharides extracted by conventional hot-water methods (15.95% residue reported [48]). The TGA results demonstrated that the enzymatically prepared MAPs possess considerable thermal stability, supporting their potential suitability for applications involving thermal processing in the food industry.

### 3.2. Protective Effects of MAPs on Alcohol-Treated N2a Cells

**Figure 2:**
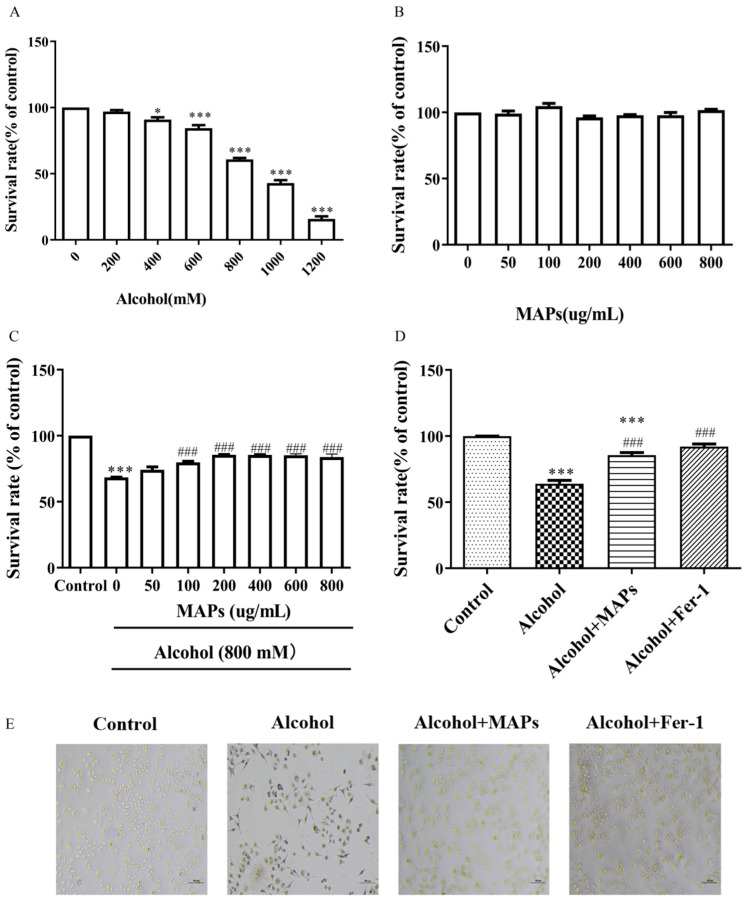
The effect of MAPs on cell viability of N2a cells. (**A**) Cell viability of N2a cells treated with alcohol. (**B**) Cell viability of N2a cells treated with MAPs. (**C**) The effect of different concentrations of MAPs on the viability of N2a cells treated with alcohol. (**D**) Cell viability of N2a cells treated with alcohol (800 mM), MAPs (200 μg/mL) and Fer-1 (10 μM)_, respectively. (**E**) Cell morphology images with different treating. Statistical significance: * *p* < 0.05, *** *p* < 0.001 vs. control group; ### *p* < 0.001 vs. alcohol group.

To evaluate the neuroprotective potential of MAPs, an ethanol-induced injury model was first established in N2a cells. The MTT-based cell viability assay elucidated that exposure of N2a cells to graded concentrations of alcohol resulted in a dose-dependent reduction in cellular proliferation and cell viability. 800 mmol/L ethanol significantly reduced cell viability to 60.85% of the control (F(6,14) = 331.0, *p* < 0.0001; Figure 2A), accompanied by morphological signs of damage. While the use of an 800 mM ethanol model was essential for the present study to establish a robust and well-defined cellular injury platform—allowing for the clear elucidation of the MAPs’ protective effects and their associated mechanism—we acknowledge its inherent limitation regarding physiological relevance. This high-concentration, acute exposure paradigm does not directly recapitulate the conditions of chronic alcohol consumption in vivo. Our experimental data (see Figure S1), showing that a lower, more clinically relevant dose of 100 mM ethanol induced a milder injury phenotype where the MAPs’ effects did not reach statistical significance, underscores the context-dependent nature of this protective activity. This observation, consistent with similar high-dose approaches commonly employed in vitro for mechanistic screening across various cell types [49,50,51,52,53,54,55], highlights a fundamental trade-off between model robustness and physiological fidelity. Future studies are imperative to validate the neuroprotective potential of MAPs in relevant models, such as those employing chronic, low-dose ethanol exposure or in vivo animal models of alcohol-induced neurotoxicity. Such investigations will be crucial for bridging the gap between our promising in vitro mechanistic findings and potential therapeutic applications.

While MAPs alone (50–800 μg/mL) were non-toxic (Figure 2B), they effectively rescued ethanol-impaired viability across the tested concentration range (Figure 2C). Notably, the protective efficacy saturated at MAPs concentrations exceeding 200 μg/mL (F(7,16) = 54.99, *p* < 0.0001). Consequently, 200 μg/mL was chosen as the optimal concentration for further experiments. To elucidated the potential molecular mechanism underpinning alcohol-induced cytotoxicity, ferroptosis inhibitor Ferrostatin-1 (Fer-1) was employed. Fer-1 demonstrated a marked restoration of cell viability with a highly significant statistical difference (F(3,8) = 59.18, *p* < 0.0001; Figure 2D). This finding suggested that alcohol reduces cell viability by promoting ferroptosis.

### 3.3. Effects of MAPs on Alcohol-Induced Antioxidative Enzyme Activities

**Figure 3:**
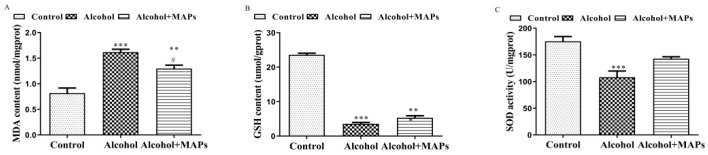
Effects of MAPs on alcohol-induced antioxidative enzyme activities. (**A**) Detection of MDA content in cells of each group. (**B**) Detection of GSH content in cells of each group. (**C**) Detection of SOD cactivity in cells of each group. ** *p* < 0.01, *** *p* < 0.001 vs. control group; # *p* < 0.05 vs. alcohol group.

Oxidative stress is one of the important means by which alcohol damages nerve cells. Normally, there is a balance between the production and removal of free radicals in the body. When alcohol acts on brain tissue, the generation of free radicals in neuronal cells surges, consuming cellular antioxidant enzymes and antioxidant substances. Excess free radicals also attack CNS membrane phospholipids to cause peroxidative damage, generating a large number of lipid peroxides (e.g., MDA), which in turn inhibit antioxidant enzyme activity [56,57]. Moreover, its metabolite acetaldehyde can also inhibit mitochondrial fatty acid oxidation, leading to lipid peroxidation, which in turn inhibits the biosynthesis of GSH. The antioxidant effect of GSH, combined with the excess free radicals produced by alcohol, led to a further reduction in glutathione content [58]. Therefore, intracellular MDA production, GSH content and SOD activities were measured (Figure 3). A marked elevation in MDA content was observed in ethanol-treated N2a cells (1.62 ± 0.74) relative to control cultures (0.82 ± 0.06), demonstrating statistical significance (F(2,4) = 25.49, *p* < 0.0001). Conversely, a notable reduction in MDA content was observed following MAPs treatment (*p* = 0.035). Ethanol exposure markedly depleted intracellular GSH (3.57 ± 0.69 vs. 23.59 ± 1.19, F(2,13) = 515.3, *p* < 0.0001) and significantly suppressed SOD activity (108.15 ± 16.46 vs. 175.40 ± 12.88 in controls, F(2,6) = 14.67, *p* = 0.0049) in N2a cells. Cotreatment with MAPs effectively attenuated these alterations, restoring GSH levels (5.32 ± 1.02, *p* = 0.135 vs. alcohol) and elevating SOD activity (142.93 ± 5.05, *p* = 0.0698 vs. alcohol). This antioxidant efficacy, evidenced by the enhanced activities of SOD and GSH, suggest that MAPs mitigate oxidative injury caused by ethanol through enhancing endogenous antioxidant mechanisms. This regulatory effect has also been demonstrated in other cells; Li et al. found that MAPs could regulate GSH and ROS levels in alloxan-induced INS-1 cells [12].

### 3.4. Effects of MAPs on Alcohol-Induced Ferrotosis of N2a Cells

**Figure 4:**
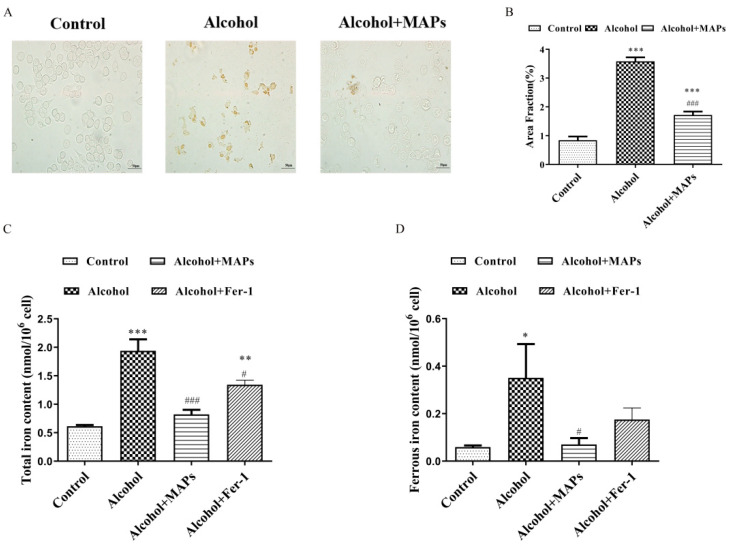
Effects of MAPs on alcohol-induced ferrotosis of N2a cells. (**A**) Iron staining of N2a cells. (**B**) Statistical results of iron staining of N2a cells. (**C**) Detection of total iron content in cells of each group. (**D**) Detection of ferrous iron content in cells of each group. * *p* < 0.05, ** *p* < 0.01, *** *p* < 0.001 vs. control group; # *p* < 0.05, ### *p* < 0.001 vs. alcohol group.

Previous studies have shown that increased iron levels can lead to oxidative stress and exacerbate neuronal degenerative death by interacting with oxygen free radicals. The cell death pathway regulated by ferroptosis is a key cell death pathway in neurons [59]. Intracellular iron accumulation is a critical event in initiating ferroptosis. This dysregulation can stem from enhanced cellular uptake, impaired iron export, or compromised storage capacity, collectively leading to a state of iron overload that consequently promotes ferroptotic cell death. Therefore we used iron staining combined with DAB enhancement to observe iron deposition in N2a cells. As shown in Figure 4A, the alcohol group showed obvious brown precipitation, which confirmed the presence of ferroptosis in this model; while the brown precipitation was significantly reduced by the administration of MAPs (F(2,6) = 115.9, *p* < 0.0001; Figure 4B). Consistent findings were obtained in measurements of cellular total iron (F(3,11) = 24.7, *p* < 0.0001; Figure 4C) and ferrous iron content (F(3,14) = 4.94, *p* = 0.0152; Figure 4D). These findings indicate that alcohol promotes intracellular iron deposition, while MAPs can counteract ferroptosis-related pathways in alcohol-induced iron metabolism dysregulation.

**Figure 5:**
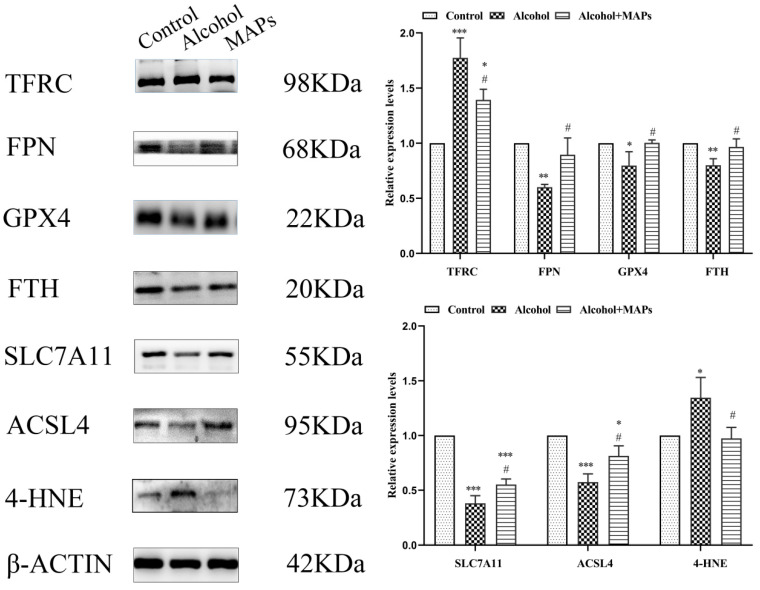
Expressions of ferroptosis-related proteins were analyzed by Western blot. * *p* < 0.05, ** *p* < 0.01, *** *p* < 0.001 vs. control group; # *p* < 0.05 vs. alcohol group.

Cellular iron homeostasis is critically regulated by specific proteins. Extracellular Fe^3+^ is internalized via transferrin receptor 1 (TFR1) and subsequently reduced to Fe^2+^ within endosomes, elevating the labile iron pool [60]. Conversely, in mammalian systems, ferroportin (FPN1) serves as the exclusive iron-exporting protein that transports ferrous ions into the systemic circulation [61]. Intracellular iron storage is primarily managed by ferritin, a supramolecular assembly comprising two distinct polypeptide chains—the ferritin light (FTL) and heavy (FTH) subunits [62]. When intracellular iron exceeds homeostatic capacity, excess Fe^2+^ can saturate ferritin storage capacity, leading to the degradation of this complex and a consequent release of free Fe^2+^, further amplifying oxidative stress. Given the pivotal roles of TFR1, FTH1, and FPN1 in maintaining iron equilibrium, we employed Western blot analysis to investigate their expression levels in our established model of iron metabolic dysregulation. As shown in Figure 5, compared with the alcohol group, the MAPs group displayed a significantly increased level of FTH1 and FPN1, a distinctly decreased TFR1 level. This suggests that MAPs inhibit ferroptosis by limiting iron uptake, promoting iron efflux, and reducing intracellular iron stores.

The main feature of ferroptosis is the accumulation of iron-dependent lipid peroxidation. The results of 3.3 indicated that MAPs regulate the activity of antioxidant enzymes such as GSH. The onset of ferroptosis results from two interconnected events. Firstly, inhibition of the system Xc^−^ transporter impairs cystine uptake and depletes the cellular pool of glutathione (GSH). This depletion, in turn, inactivates the selenoenzyme GPX4 primarily catalyzes the reductive detoxification of phospholipid hydroperoxides. The consequent accumulation of phospholipid peroxides and reactive oxygen species beyond a critical threshold drives ferroptotic cell death [63]. GPX4 is a specific enzyme that scavenges lipid peroxides via GSH and is a key regulatory molecule for ferroptosis [64]; and SLC7A11 is an upstream molecule of the GPX4 antioxidant system [65]. To investigate the potential involvement of ferroptosis, we assessed the expression of key regulatory proteins. Western blot analysis revealed that ethanol exposure markedly downregulated the protein levels of both GPX4, a central regulator of ferroptosis, and SLC7A11, the functional subunit of system Xc^−^, compared to the control group (Figure 5). Conversely, treatment with MAPs significantly elevated their expression, indicating a restorative effect on this critical antioxidant defense pathway. It suggested that MAPs resisted the onset of ferroptosis by activating the activity of GPX4 and up-regulating system XC^−^.

On this basis, we also assessed the level of lipid peroxidation by Western blot detection of ACSL4 and 4-HNE protein expression. ACSL4, an important lipid metabolizing enzyme, is involved in ferroptosis by converting free Arachidonic acid (AA) to arachidonic aldehyde-CoA to generate lipid hydroperoxides; whereas, as a characteristic molecular marker indicative of peroxidative damage and disrupted redox homeostasis, the large accumulation of 4-HNE is one of the key factors driving the ferroptosis process [66]. As shown in Figure 4B, protein abundance of established indicators for lipid peroxidation (ACSL4 and 4-HNE) was assessed. They were markedly elevated in the alcohol-treated group relative to the control, whereas expression levels of these two pivotal biomarkers were significantly decreased after the intervention of MAPs. It suggested that MAPs prevented the accumulation of lipid peroxides and thus inhibited ferroptosis.

Our findings demonstrated that MAPs can ameliorate ethanol-induced neuronal damage by inhibiting ferroptosis pathways in vitro. But the high molecular weight of MAPs presented a well-recognized challenge for direct penetration of the blood–brain barrier (BBB), a common hurdle for polysaccharide-based neuroprotective agents. As evidenced by prior research on other high-molecular-weight polysaccharides, such as konjac glucomannan and Gastrodia elata polysaccharides, systemic neuroprotection can be achieved through indirect pathways like modulating the gut–brain axis [67,68], which represents one viable translational route for MAPs. Concurrently, the inherent biocompatibility and ease of functionalization of natural polysaccharides had positioned them as excellent candidate materials for constructing brain-targeted nanodelivery systems in contemporary research [69,70]. Building on this, we propose a hypothesis: the MAPs characterized in this study could be engineered into a brain-targeted nanodelivery platform. By modifying their surface with BBB-transcytosis ligands (e.g., targeting the transferrin receptor), MAPs-based nanoparticles could be designed to encapsulate and ferry small-molecule neuroprotective drugs across the BBB, thereby overcoming the inherent delivery challenge. Future work will focus on evaluating the in vivo efficacy of MAPs via systemic administration and on the rational design and validation of such multifunctional MAPs-derived nanocarriers.

## 4. Conclusions

As one of the most important functional components of cactus, the structure of MAPs is numerous and complex. MAPs have various biological functions, such as neuroprotective, antioxidant and antitumor activities, and are affected by different aspects of the polysaccharides themselves. This study investigated the MAPS extracted via enzymatic methodology consisting of nine different monosaccharides and glycoalkaloids. MAPs are homogeneous heteropolysaccharides with an average molecular weight of 8.79 × 10^6^ Da. MAPs are found in combination with sulfated polysaccharides and glycoalkaloids, and contain both α-glycosidic and β-glycosidic bonds with an overall low crystallinity and favorable thermal stability.

Alcohol causes a series of adverse reactions to the nervous system. MAPs have been found to restore nerve alcoholic cell damage by reducing iron deposition, enhancing antioxidant capacity and inhibiting lipid peroxidation. These results suggested that the protective effect of MAPs on alcoholic nerve injury is possibly related to cellular ferroptosis.

## Figures and Tables

**Table 1:** Chemicals analysis and monosaccharide composition of *Opuntia milpa alta* polyaccharides.

	MAPs	
Neutral sugar (%)	74.50 ± 0.21	
Uronic acid (%)	11.43 ± 0.18	
	Monosaccharide composition (mol%)	Estimated number of residues per molecule
Mannose	7.11 ± 0.15	~4067
Ribose	4.01 ± 0.10	~2294
Rhamnose	19.69 ± 0.33	~11,260
Glucuronic acid	3.95 ± 0.12	~2259
Galacturonic acid	12.08 ± 0.42	~6910
Glucose	2.93 ± 0.09	~1676
Xylose	8.74 ± 0.18	~5000
Galactose	14.73 ± 0.19	~8425
Arabinose	26.77 ± 0.32	~15,310

## Data Availability

The original contributions presented in this study are included in the article/Supplementary Materials. Further inquiries can be directed to the corresponding authors.

## Supplementary Materials

The following supporting information can be downloaded at: https://www.mdpi.com/article/10.3390/foods15020249/s1, Figure S1. The total iron and ferrous ion contents of each group at an alcohol concentration of 100 mM.
