# Supplementary material for: Polysaccharides Extraction from Opuntia milpa alta and Their Protective Effect on Alcohol-Induced Neuro 2a Cell Damage via Ferroptosis
Source: Foods. 2026 Jan 9;15(2):249. doi: 10.3390/foods15020249 (PMC12840309; doi:10.3390/foods15020249)

Figure S1. The total iron and ferrous ion contents of each group at an alcohol concentration of 100 mM

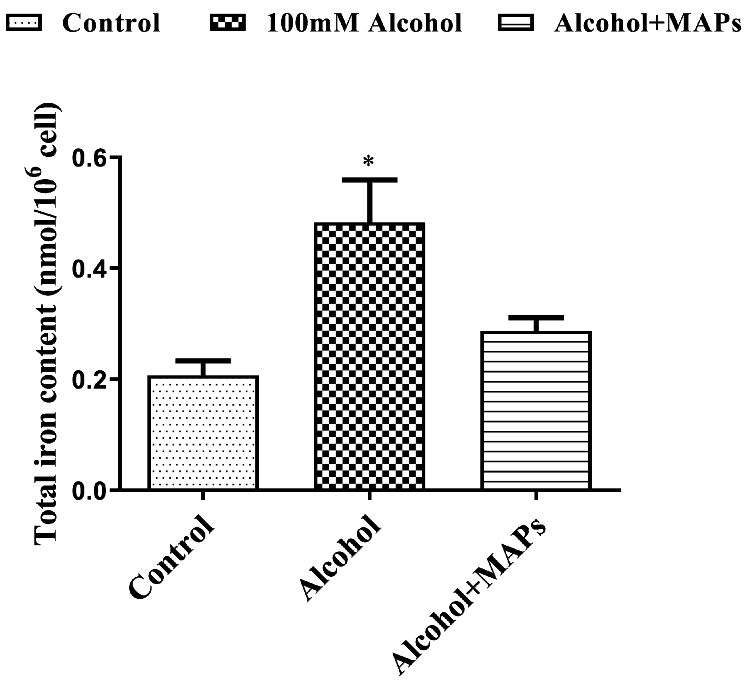

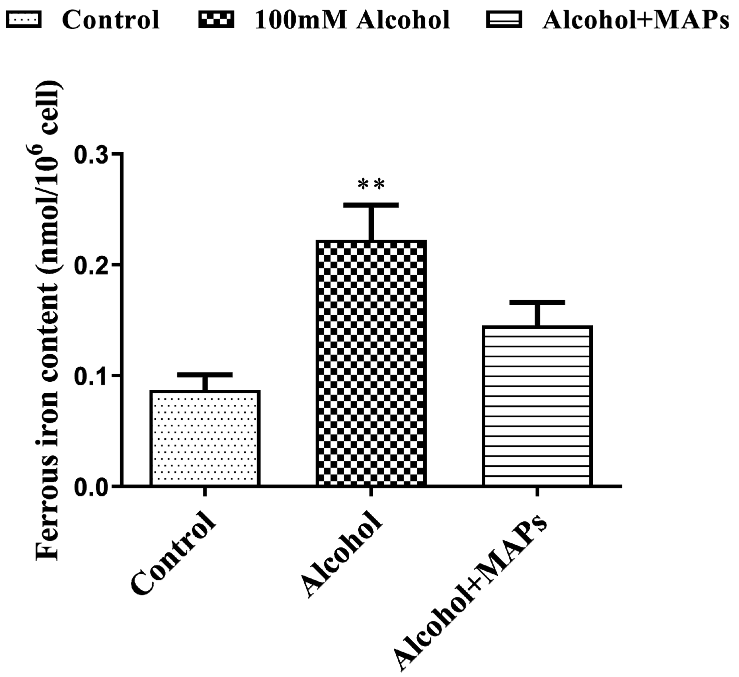

Supplement: Supplementary file 1 [file foods-15-00249-s001.zip › foods-4011249-supplementary.pdf]
